# The role of muscle ultrasound in helping the clinical diagnosis of muscle diseases

**DOI:** 10.1186/s41983-018-0039-6

**Published:** 2018-11-01

**Authors:** Hanan Helmy, Ahmed Aboumousa, Asmaa Abdelmagied, Aya Alsayyad, Sandra Ahmed Nasr

**Affiliations:** 1 Neurology, Cairo University, Cairo, Egypt; 2 Radio-Diagnosis, Cairo University, Cairo, Egypt

**Keywords:** Myopathy, Muscle ultrasound, Muscle MRI

## Abstract

**Background:**

Selective involvement of certain muscles is an indicator for muscle diseases and helps to direct the diagnosis, but in some cases, it cannot be detected clinically; hence, the roles of muscle MRI and ultrasound are to detect this selectivity and facilitate the diagnosis.

**Objectives:**

The possibility of using muscle ultrasound as a screening tool when muscle diseases are suspected and as an alternative to MRI.

**Subjects and methods:**

This cross-sectional descriptive study included 38 patients presented with clinical manifestations suggestive of muscle diseases. The patients were selected over a period of 1 year. All patients were subjected to thorough clinical assessment and muscle ultrasound of the thigh and leg for all patients, while 15 were subjected to MRI. Clinical and radiological assessments were performed separately, followed by both clinical and radiological findings to assess the power of combining the clinical and radiological assessments for the diagnosis of muscle diseases.

**Results:**

The clinical assessment reached a main provisional probable diagnosis in 53% cases, and radiological assessment blind to clinical data suggested diagnosis in 18 of the total cases, while the combination of both ultrasound and MRI could suggest diagnosis in 87% of the cases. The concordance ratio of ultrasound to MRI ranged between 78 and 100%.

**Conclusion:**

The combination of clinical and radiological assessments of muscle diseases can suggest a main provisional probable diagnosis, especially when genetic diagnosis is not accessible, or to direct the genetic testing when it is available. Ultrasound can be used as a routine tool in screening and follow-up of muscle diseases.

## Introduction

Many muscle diseases share common clinical features that render arriving at appropriate differential diagnoses difficult. The selective involvement of certain muscles is a key to the diagnosis of muscle diseases. Muscle MRI can detect the pattern of muscle affection, especially when clinical detection is difficult**.** The combination of muscle imaging with clinical can limit the differential diagnosis and even yield the most probable one and can direct genetic testing as the only method to arrive at a definite diagnosis [1].

High-resolution ultrasound allows the visualization of the muscle, nerve, and adjacent structures and can offer real-time information in neuromuscular diseases [2]. Additionally, it entails a safe, accessible, low-cost, and no ionizing radiation, with no known contraindications and has no problems associated with MRI such as claustrophobia, metallic implants, and the need for sedation in children; therefore, it can be used as a complementary tool to electro-diagnosis [3]. However, its main disadvantage is it is operator-dependent, with limited penetration in the imaging of deeper structures [4].

The aim of this study is to detect the role of combining clinical and radiological assessments in the diagnosis of muscle diseases and the possibility of using muscle ultrasound as a screening tool when muscle diseases are suspected and as alternative to MRI.

## Patients and methods

This is a cross-sectional descriptive study that included 38 patients, presented with clinical manifestations suggestive of muscle disease according to the clinical picture and confirmed by electromyography (EMG) and possibly CPK serum level.

Patient’s history included the age of onset, disease progression, family history, and history of cardiac or respiratory affection. Full general examination was done. Neurological examination included the presence of cranial nerve affection, assessment of the muscle state (atrophy, hypertrophy), presence of contractures, detection of selective muscle affection and distribution of weakness, and clinical assessment of gait patterns (heal strike, tiptoe walking, foot drop, and waddling gait). In the assessment of muscle power through Medical Research Council (MRC) grading (muscle strength grading system by the Medical Research Council), patients were divided into three groups according to their first presentation, delayed motor milestones, limb girdle weakness, and with prominent cranial musculature involvement.

### Ultrasound examination

Muscle ultrasounds were performed using Philips HDI5000, brand ATL, model HTI 5000, and Origin USA linear probe 5–12 MHz. The probe was used in a transverse plane, perpendicular to the long axis of the muscle. The muscle was examined though its whole length dynamically inspected for any spontaneous activity at rest. The following the muscles were inspected: (a) anterior thigh: rectus femoris (RF), vastus intermedius, lateralis and medialis, sartorius, and gracilis; (b) posterior thigh: adductors, biceps femoris, semitendinosus, and semimembranosus; (c) leg: tibialis anterior, peronii, and gastrocnemius soleus.

We employed a visual grading system to detect whether the muscles were affected. Normal muscle appears with low echo-intensity divided by echogenic perimysium and connective tissue with speckled appearance (muscle striation) in the transverse plane. The muscle was considered abnormal if it showed increased echogenicity, lost its striation, or showed change in muscle bulk (atrophy or hypertrophy).

Muscle selectivity was determined and described if present.

### MRI examination

MRI was done for a sub-group of 15 patients for both thighs and legs by the use of Philips Interna 1.5T, Philips, Achieva 1.5T, and GE Signa 0.2T systems. MRI cuts included are the axial section of conventional T1- and T2-weighted sequences, for the evaluation of muscles’ signal intensity and bulk. The muscle was considered abnormal if it showed high signal intensity with loss of muscle striation. We utilized the pattern of selective muscle involvement described by Abe and his colleagues (2016) [5].

This study was approved by the ethical committee of the faculty of medicine, Cairo University.

## Statistical analysis

This is a descriptive study that the collected data were coded, tabulated, and statistically analyzed using IBM SPSS Statistics (Statistical Package for Social Sciences) software version 22.0, IBM Corp., Chicago, USA, 2013.

## Results

A total of 38 patients were included in this study. The patients were divided into 3 clinical groups according to clinical presentation: (a) delayed motor milestones, (b) limb girdle weakness, and (c) with prominent cranial nerves involvement Table 1.

**Table 1:** Clinical features of patients

Clinical type	Number of patients	Age of patients (range)	Age of onset (range)	Duration of disease (range)	Calf muscle state		
Delayed motor milestones	11	4–30 years	Floppy infant since birth - up to 4 years of age	3–28 years	Preserved	Hypertrophied	Wasted
					6 patients	5 patients	0 patients
Limb girdle weakness	17	13–43 years	10–40 years	6 months–22 years	8 patients	6 patients	3 patients
With prominent cranial musculature involvement	10	13–40 years	4–20 years	6–26 years	10 patients	0 patients	0 patients

Patients with delayed milestones (*n* = 11) included 3 females and 8 males. Four patients were diagnosed as definite Duchenne muscular dystrophy and one with definite myotonic dystrophy. Other patients in this group fell in the spectrum of other dystrophies and congenital myopathies. Muscle involvement in ultrasound was specific and suggestive of a probable possible diagnosis in 4 patients without clinical data, as the ultrasound results detected increased connective tissue, suggesting the presence of connective tissue disease or connective tissue-related myopathy, preferential affection of the vastus intermedius (VI), sparing other vasti giving characteristic semilunar pattern of myotonic dystrophy, and quadriceps-sparing myopathy. Ultrasound confirmed the diagnosis of myotonic dystrophy, also Duchenne muscular dystrophy, detecting a greater affection of the anterior compartment than the posterior one in the thigh, along with the affection of the adductors, leg affection of tibialis anterior, and of both heads of gastrocnemius more than soleus.

**Fig. 1:**
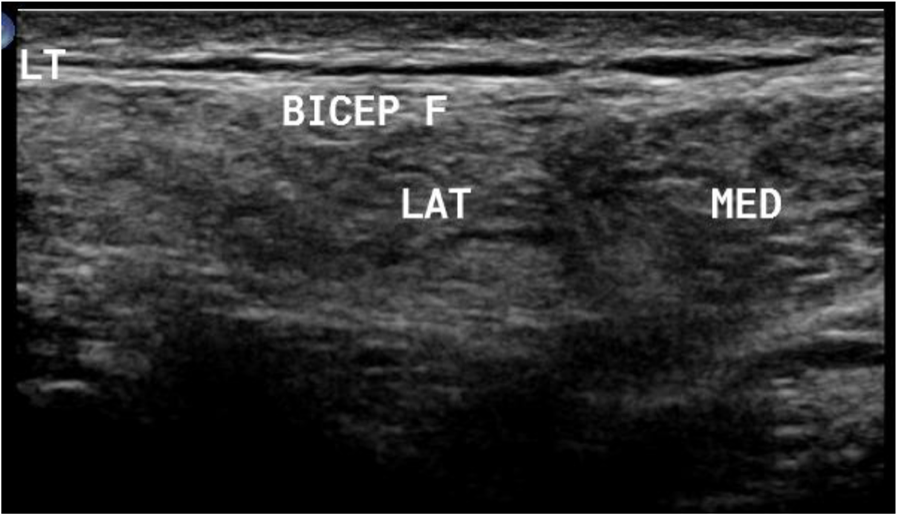
In patient with LGMD2B, ultrasound detected preferential affection of both heads of biceps brachii

**Fig. 2:**
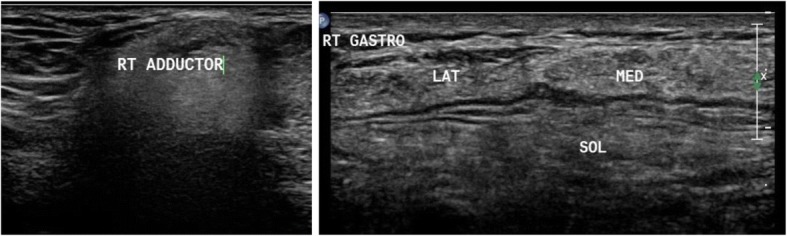
Muscle ultrasound in patient with limb girdle muscular dystrophy 2A (LGMD2A) showing in the thigh more affection of posterior compartment with predominant affection of the adductors, in the leg more affection of medial head of gastrocnemius (MED GASTRO) and soleus (SOL) more than lateral head with relative sparing of tibialis anterior (TA)

**Fig. 3:**
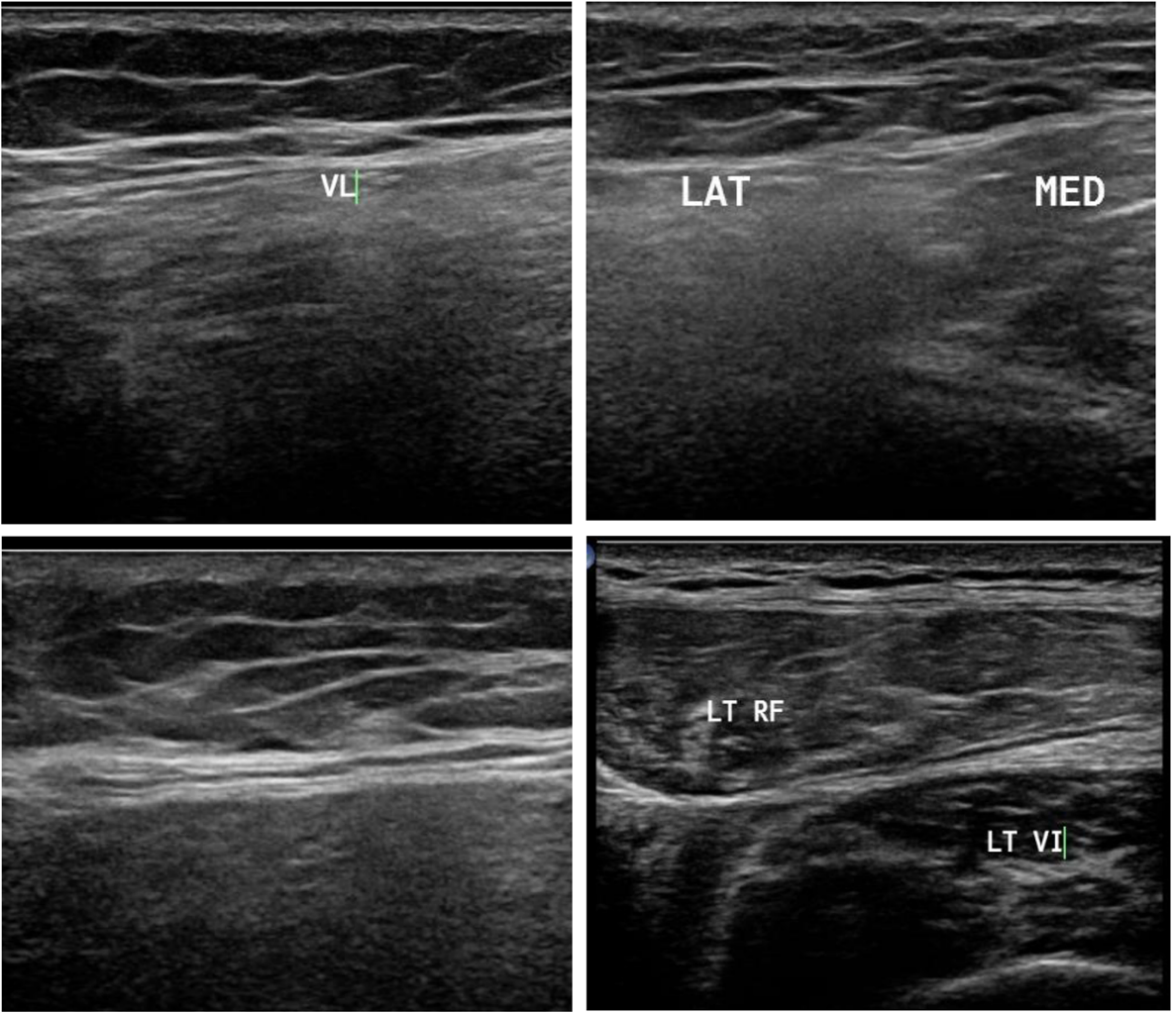
Muscle ultrasound in patient with X-linked recessive Emery-Dreifuss muscular dystrophy (EDMD) showing minimal affection in the thigh especially of the vastus lateralis (VL) and vastus intermedius (VI) and in leg selective affection of soleus

**Fig. 4:**
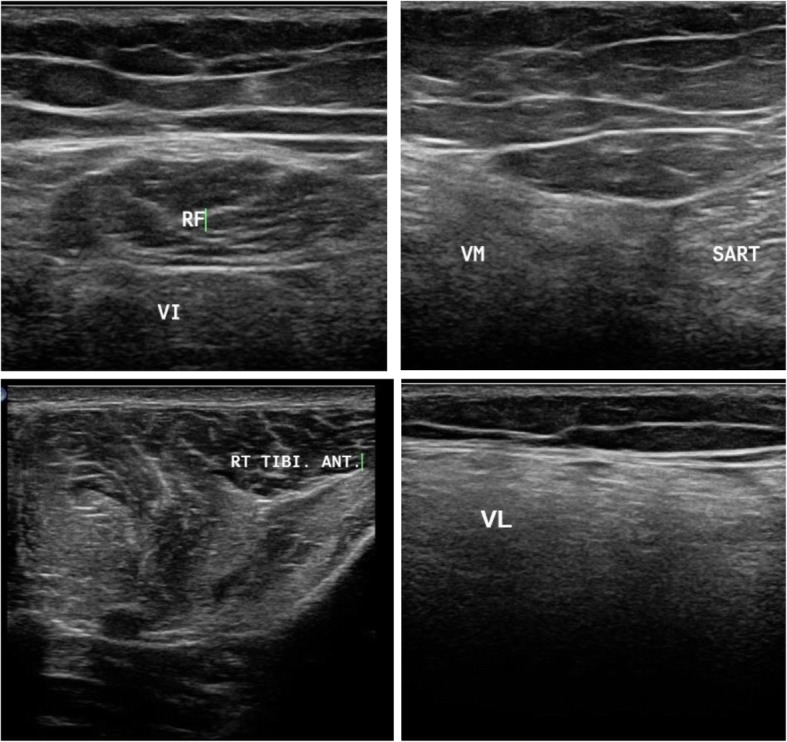
Muscle ultrasound in patient suspected to be with acid maltase deficiency showing affection of the vastus intermedius (VI) and sparing of the rectus femoris (RF) and vastus lateralis (VL) and affection of the vastus medialis (VM) with sparing tibialis anterior (TA)

Patients with limb girdle weakness (*n* = 17) included 4 females and 13 males. One patient was diagnosed with rippling muscle disease due to the presence of myokymia. One patient had symptoms of polymyositis and another one of dermatomyositis. Other patients fell in the spectrum of limb girdle muscle dystrophy. In this group, the presence of moth-eaten appearance and fasciculation-directed diagnosis towards neurogenic diseases was observed. Ultrasound confirmed the diagnosis of LGMD2B when it detected a greater affection of the vastus lateralis and the affection of both heads of gastrocnemius and selective affection of one head of biceps brachii, sparing the other one, as depicted in Fig. 1. Idiopathic inflammatory myopathy showed a diffuse pathology with no selectivity improvement post treatment. Ultrasound detected myokymia at rest in rippling muscle disease. It reached only one most probable diagnosis in LGMD2A due to greater affection in the thighs in the posterior than the anterior compartment, with the predominant affection of the adductors, and in the legs, greater affection of medial head of gastrocnemius and soleus, more than the lateral head, with relative sparing of tibialis anterior, as depicted in Fig. 2. LGMD2I ultrasound finding showed leg affection mainly in the medial head of gastrocnemius and soleus more than the lateral head, with the sparing of anterior compartment including the tibialis anterior. Becker muscle dystrophy showed greater leg affection of gastrocnemius than soleus. X-linked recessive EDMD was suggested due to minimal affection in the thigh, especially of the vastus lateralis and VM, and in leg selective affection of soleus, as illustrated in Fig. 3. Patients with spinal muscular atrophy had visualized fasciculations and moth-eaten appearance of the affected muscles. It helped us in the diagnosis when it is difficult to make it clinical, as in the case of acid maltase, deficiency myopathy ultrasound revealed affection of the vastus intermedius (VI) and vastus medialis with sparing of the rectus femoris and vastus lateralis (VL), with no differential affection of both heads of gastrocnemius and soleus with sparing anterior leg, as illustrated in Fig. 4, and in the upper limb, the biceps brachii was affected more than the triceps. Furthermore, it helps us when secondary myopathy is suspected as the muscles appear normal.

Patients with prominent cranial musculature involvement (*n* = 10) involved 2 females and 8 males. The clinical presentation facilitated arriving at a main provisional probable diagnosis for all patients. This group included patients with clinically diagnosed FSHMD, myotonic dystrophy, and congenital myasthenia. Myotonic dystrophy patients showed a semilunar pattern and delayed muscle relaxation (myotonic phenomenon). The ultrasound result was normal for congenital myasthenia patients. In FSHMD with the absence of lower limb weakness, the ultrasound showed no abnormalities. In more advanced FSHMD patients, asymmetrical affection was found.

According to the state of the calf muscles, ultrasound confirmed the suggested clinical diagnosis in patients with hypertrophied or atrophied calf muscles. Yet, it showed less accurate finding in patients with preserved calf muscles Table 2.

**Table 2:** Classification of patients according to calf state and its relation to clinical and ultrasound diagnosis

Calf state	Number of patients	Reaching most probable diagnosis clinically	Ultrasound proved or added to diagnosis
Calf hypertrophy	11 patients (29%)	6 patients (54.5%)	11 patients (100%)
Calf wasting	3 patients (8%)	1 patient (33.3%)	3 patients (100%)
Preserved	24 patients (63%)	12 patients (50%)	20 patients (83.3%)

In this study, the clinical assessment reached a main provisional probable diagnosis in 53% of the cases and the radiological assessment blind to the clinical data suggested the diagnosis in 18% of the cases, while the combination of both could suggest the diagnosis in 87% of the cases. The concordance ratio of ultrasound to MRI ranged between 78 and 100% Table 3.

**Table 3:** Clinical and radiological diagnosis and relation between both

Clinical diagnosis		Ultrasound diagnosis
Provisional diagnosis	20 patients (53%)	Confirmed the diagnosis in these 20 patients (100%)
2 or 3 diagnosis	10 patients (26%)	Limited the D.D in 8 patients (80%)
Failed clinical diagnosis	8 patients (21%)	Directed diagnosis in 5 patients (63%)
Without clinical data		Ultrasound can suggest diagnosis in 7 patients (18%)

In our study, we found that the long duration of an illness is not an obstacle in the detection of the selectivity of affected muscles by ultrasound. There was no relation between the level of CPK and ultrasound findings. Weaker muscles had not necessarily more echo-intensity than other muscles.

## Discussion

As muscle diseases share common presentation and selectivity in muscle affection, they may not be detected clinically; so, it is important to combine both clinical and radiological assessment to reach the most probable diagnosis [6], to guide genetic counseling, cardio respiratory risk evaluation, prognostic assumption, and future therapeutic possibilities [7].

Ultrasound can detect the same patterns of muscle affection as MRI [8], it has no known contraindications and can provide real-time information related to muscle activation and movement patterns; so, selective substitution of muscle ultrasound for MRI can result in significant cost-saving for the health care system [9].

This study aimed to describe the effect of combining both clinical and radiological assessments to limit the differential diagnosis and reach the most probable one in patients with picture suggestive of muscle diseases. Also, we aimed to assess the role of ultrasound as an initial screening tool in evaluation of muscle diseases as an alternative to MRI.

The clinical assessment could reach a main provisional probable diagnosis in certain cases, for instance, in Duchene muscular dystrophy (DMD), since the clinical presentation is rather classic: young boys with delayed motor milestones, calf hypertrophy, and positive Gower’s sign [10]. Limb girdle dystrophy 2B (LGMD2B) (dysferlin) presents with characteristic heal strike and prominent weakness and atrophy of one head of biceps brachii [6]. Myotonic dystrophy (DM) characteristically has positive myotonic phenomenon [11]. FSHMD has facioscapulohumeral weakness with positive Beevor’s sign [12]. Polymyositis presents with sub-acute onset of dyspnea, muscle pains, and bulbar symptoms. Dermatomyositis has additional skin lesions [13].

The clinical assessment was sometimes not helpful, may be due to the absence of selective muscle affection, as in cases of delayed presentation or early presentation before the appearance of any clinical clues, such as in cardiac or respiratory affection [6]. Ultrasound can detect focal or patchy abnormalities within muscle groups, as in the affection of the part of a muscle that spares the other one, and can be performed quickly and at the patient’s bedside; so, it could be added to the routine evaluation of muscle diseases [14].

We compared ultrasound results with MRI for 15 patients (40%); the concordance ratio of ultrasound ranged from 78 to 100%, it was 100% in 10 patients (67%). It was performed safely in the case of floppy infants, and the moth-eaten appearance could differentiate spinal muscular atrophy (SMA) from myopathy [4]. We found that ultrasound was as informative as an MRI as it was previously stated [8].

Ultrasound with or without MRI confirmed the clinical diagnosis in 20 patients and added to the diagnosis for the other 13 patients; so, it helped in the diagnosis of 33 patients (87%). It can detect quadriceps sparing myopathy, selective affection of one head of gastrocnemius as of medial head of gastrocnemius and soleus more than lateral head in FKRP (LGMD2I) and calpain-3 deficiency (LGMD2A), and selective affection of lateral head of gastrocnemius as in LGMD2B (dysferlin) [15].

In patients with early contractures, the clinical assessment produced a differential diagnosis of LGMD2A, Bethlem myopathy, and EDMD [16]. The ultrasound and MRI can differentiate between them as the selective affection of the medial head of gastrocnemius and soleus more than the lateral head in calpain-3 deficiency (LGMD2A), selective involvement of the soleus in x-linked EDMD [17], and affection of the periphery of the gastrocnemius and soleus muscle, showing a rim in between them and the special affection of the middle of the rectus femoris muscle, and the central shadow sign in Bethlem myopathy [18].

In patients with limb girdle pattern of weakness with calf hypertrophy, the ultrasound helped the diagnosis by detecting the affection of the medial head of gastrocnemius and soleus more than the lateral head in FKRP (LGMD2I), greater affection of gastrocnemius than soleus in Becker muscular dystrophy (BMD). So, in the case of preferential affection in calf muscles that cannot be detected clinically, both MRI and ultrasound and even ultrasound alone detected differences between both heads of gastrocnemius and gastrocnemius and soleus [15].

Also, in myotonic dystrophy, a characteristic perifemoral semilunar pattern of affection was found [1]. In peripheral floppy infant patients, the ultrasound was used safely without the risk involved in anesthesia as in MRI; it helped to narrow down the differential diagnosis, as it differentiated between whether it was caused due to myogenic or neurogenic disease and whether the echogenicity was homogenous as in myogenic diseases or inhomogeneous as in SMA [19]. Based on the selectivity of muscle affection, it differentiated between different types of congenital myopathies and congenital muscular dystrophy (MD), congenital myasthenia, and connective tissue (CT) disease, or CT-related myopathy. In one patient with myopathy with limb girdle pattern of weakness, imaging detected nearly normal muscle signal that pushed us to search for a secondary myopathy, and we found an elevated PTH level. When an adult onset SMA presented with an LGM-like clinical picture, with CK elevation, ultrasound differentiated SMA from myopathy by the presence of moth-eaten appearance; also, it detected the presence of fasciculation. Further, the ultrasound detected the presence of myokymia in patients with limb girdle weakness and calf hypertrophy, directing the diagnosis towards rippling muscle disease. There was a group of undiagnosed patients; in this group, radiological assessment did not add suggestions for diagnosis, which may be due to diffused muscular fatty infiltration or lack of specific patterns of muscular affection [6].

In our study, we found that the long duration of illness may not be an obstacle in detecting selectivity of affected muscles, as in advanced BMD, less affection of soleus, more than the gastrocnemius was observed, and even after more than 20 years, imaging detected quadriceps sparing myopathy, and more affection of lateral head of gastrocnemius in LGMD2B. However, in some patients, diffused affection and lost selectivity was also observed; also, we found no relation between the ultrasound findings and the age of patients, similar to Zaidman and his colleagues’ (2011) findings [14]. This is in contrast to Trip and colleagues (2009), who found an increase in echo-intensity in the muscles with age, due to age-related replacement of muscle tissue by fat and fibrosis [20].

We found no relation between the level of CPK and ultrasound findings, as in DMD, CPK may be markedly elevated, but the ultrasound still could detect more affection of the muscles of anterior compartment and adductors, and in FSHMD, when CPK was usually normal, diffused affection of the muscles was observed, and asymmetrical affection formed our indicator.

So, the level of CK will not limit the use of ultrasound. Tieleman and colleagues (2012) found no relation between muscle echo-intensity and CPK level [21].

It was not necessary to find that weaker muscles had more echo-intensity than other muscles, as an ultrasound may detect more affection in the muscles which could not be detected clinically. In contrast to our results, Jansen and colleagues (2012), in a study on boys with DMD, found that there was a negative correlation between the echo-intensity of the muscles and the muscle strength [7].

In our study, we found that when FSHMD was suspected clinically, there was no need to do an ultrasound, as most of the patients reported the pain after a long duration, so the selectivity was lost from the asymmetrical affection that was detected clinically; therefore, we will not recommend ultrasound for patients with FSHMD.

Ultrasound can be used for follow-up of patients, especially when MRI cannot be done due to the presence of pacemaker or severe pulmonary problems [21], also when there is a risk involved with anesthesia as when congenital myopathies are suspected [22].

Ultrasound can detect changes in the muscle with regard to disease progression or change in the muscles due to treatment; so, it can be used to follow-up on the disease progression and results of treatment [21].

In conclusion, combination of clinical and radiological assessments of muscle diseases can suggest a main provisional probable diagnosis, especially when genetic diagnosis is not accessible or expensive, or to provide a guide to the proper genetic testing when it is available. Ultrasounds can be used as a routine tool to screen and follow-up on muscle diseases and as alternative to MRI.

## Abbreviations

BMD: Becker muscular dystrophy
CPK: Creatine phosphokinase
CT: Connective tissue
DD: Differential diagnosis
DM: Dermatomyositis
DM: Myotonic dystrophy
DMD: Duchene muscular dystrophy
EDMD: Emery-Dreifuss muscular dystrophy
EMG: Electromyography
FSHMD: Fascioscapulohumeral muscular dystrophy
LGM D2A: Limb-girdle muscular dystrophy
LGM D2B: Limb-girdle muscular dystrophy
LMN: Lower motor neuron
MRC: Medical research council
PM: Polymyositis
RF: Rectus femoris muscle
SM: Semimembranosus muscle
SMA: Spinal muscular atrophy
ST: Semitendinosus muscle
VI: Vastus intermedius muscle
VL: Vastus lateralis muscle
VM: Vastus medialis muscle
